# Impact of uniaxial strain and doping on oxygen diffusion in CeO_2_

**DOI:** 10.1038/srep06068

**Published:** 2014-08-14

**Authors:** M. J. D. Rushton, A. Chroneos

**Affiliations:** 1 Department of Materials, Imperial College London, London SW7 2AZ, United Kingdom; 2 Faculty of Engineering and Computing, Coventry University, 3 Gulson Street, Coventry CV1 2JH, UK

## Abstract

Doped ceria is an important electrolyte for solid oxide fuel cell applications. Molecular dynamics simulations have been used to investigate the impact of uniaxial strain along the <100> directions and rare-earth doping (Yb, Er, Ho, Dy, Gd, Sm, Nd, and La) on oxygen diffusion. We introduce a new potential model that is able to describe the thermal expansion and elastic properties of ceria to give excellent agreement with experimental data. We calculate the activation energy of oxygen migration in the temperature range 900–1900 K for both unstrained and rare-earth doped ceria systems under tensile strain. Uniaxial strain has a considerable effect in lowering the activation energies of oxygen migration. A more pronounced increase in oxygen diffusivities is predicted at the lower end of the temperature range for all the dopants considered.

The widespread commercial adoption of Solid Oxide Fuel Cells (SOFCs) requires their operation in the intermediate (500–700°C) temperature range^1^. This requires ceramic electrodes and electrolytes with enhanced activity, which effectively translates to higher oxygen diffusivities^2^. The SOFC community is investigating numerous candidate oxides for these applications for use in the next generation of intermediate temperature SOFCs (IT-SOFCs)^3^,^4^,^5^,^6^,^7^,^8^.

Rare-earth doped ceria is an important electrolyte material because of its high oxygen ion diffusivity and relatively low reduction temperatures^9^,^10^,^11^. In essence doping ceria with oxides such as R_2_O_3_ (here R = rare-earth) improves the oxygen diffusion due to the formation of oxygen vacancies, which act as vehicles for oxygen diffusion in the resultant Ce_1-x_R_x_O_2-x/2_ solid solution^12^. Within the intermediate temperature range, doped ceria is advantageous in comparison to more conventional fuel cell electrolytes such as yttria stabilised zirconia as it exhibits up to 2–3 orders of magnitude higher oxygen ion conductivity^13^,^14^,^15^,^16^.

Another way to increase oxygen diffusion is through strain, for example through the presence of an interface between dissimilar oxides^17^,^18^,^19^,^20^. Atomic scale modelling can provide important insights on the impact of strain and doping regimes on oxygen transport in SOFC materials^21^,^22^,^23^,^24^,^25^,^26^. Recent atomistic simulation studies were used to investigate the defect induced chemical expansion in ceria highlighting that it is important to properly model the elastic properties in doped ceria^27^,^28^.

To accurately describe oxygen transport properties the potential model must adequately describe the thermal expansion of doped ceria with respect to the rare-earth dopant. Here we have derived a new and transferable potential model that reproduces experimentally determined thermal expansion and elastic constant values for ceria with a high degree of accuracy. This has enabled the use of molecular dynamics (MD) calculations within the present study to provide reliable predictions of how uniaxial strain affects oxygen ion diffusivity as a function of temperature and rare-earth doping (Yb, Er, Ho, Dy, Gd, Sm, Nd and La).

## Results and discussion

### Model validation

This work makes use of the recently developed Cooper, Rushton and Grimes potential (CRG) model, which provides an excellent description of CeO_2_ and other fluorite oxides^29^. Distinct from similar classical models^30^,^31^, the CRG model includes many-body effects using a generalised form of the embedded atom method^32^,^33^,^34^ that allows it to successfully capture the Cauchy-violation^35^, which is necessary for accurate reproduction of the elastic constants and consequently, the strain behaviour of fluorite oxides. In addition Cooper *et al.*^29^ demonstrated that their model is capable of describing several thermo-mechanical properties such as specific heat and thermal expansion over a wide temperature range, making it suitable for use here. In particular, the model is able to reproduce the experimentally determined elastic constants of CeO_2_ with high fidelity (see Table 4 within reference 29). For the present work, the CRG model has been extended to include interactions for a number of trivalent rare-earth species typically used as dopants within CeO_2_. The derivation of the additional interactions is described within the Methods section.

**Figure 1:**
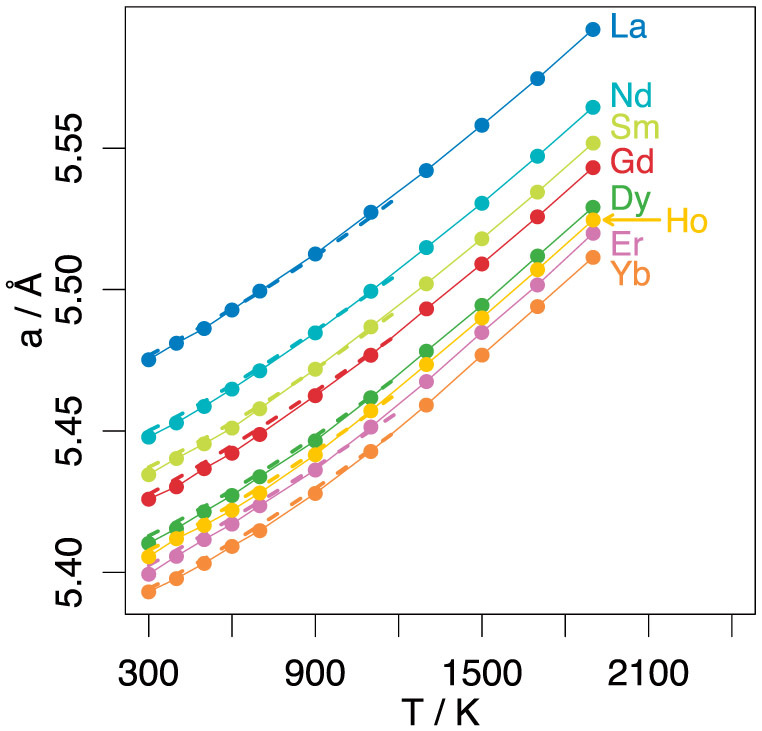
Lattice parameter as a function of temperature for doped ceria (Ce_0.8_R_0.2_O_1.9_). Solid lines show predictions made using molecular dynamics and the current potential model. The experimental data of Pikalova *et al*.^36^ is plotted for temperatures below 1200 K as dashed lines.

**Figure 2:**
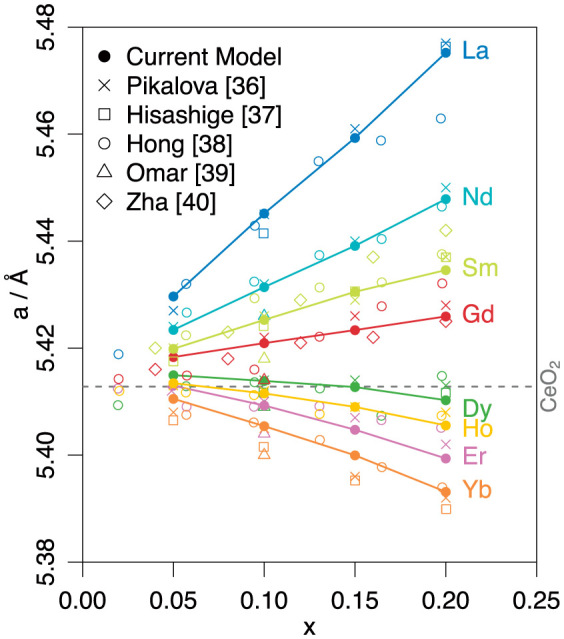
Variation in lattice parameter predicted by model for Ce_1-x_R_x_O_2-½x_ as function of dopant concentration x at T = 300 K. Molecular dynamics data is plotted with the experimental values^36^,^37^,^38^,^39^,^40^. Dashed grey line indicates the 300 K lattice parameter predicted by the model for undoped CeO_2_.

The ability of the extended CRG^29^ model to reproduce the thermal expansion of doped ceria is illustrated in Figure 1, where lattice parameter is predicted as a function of temperature for all the dopants considered and plotted alongside Pikalova *et al.*^36^ data. Whilst the dopant-oxygen potentials were derived at a fixed composition of Ce_0.8_R_0.2_O_1.9_, it can be seen from Figure 2 that the model is able to predict the lattice parameter of over a wide compositional range; this figure plots lattice parameter against *x* for all the dopants considered at a temperature of 300 K along with experimental data^36^,^37^,^38^,^39^,^40^. The predictions for the change in lattice parameter with respect to the dopant concentration obtained from the present model is in very good agreement with the experimental data^40^ with the predicted values falling well within the scatter of the experimental data.

### Trivalent doping

The introduction of the rare-earth ions, which are trivalent, leads to the oxygen deficient Ce_1-x_R_x_O_2-x/2_ solid solution. For every two rare-earth dopants introduced into the cation sublattice one oxygen vacancy is formed via the following process (given in Kröger-Vink notation^41^): 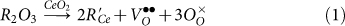
The formation of these oxygen vacancies is important as they are the vehicles for diffusion in the anion sublattice.

**Figure 3:**
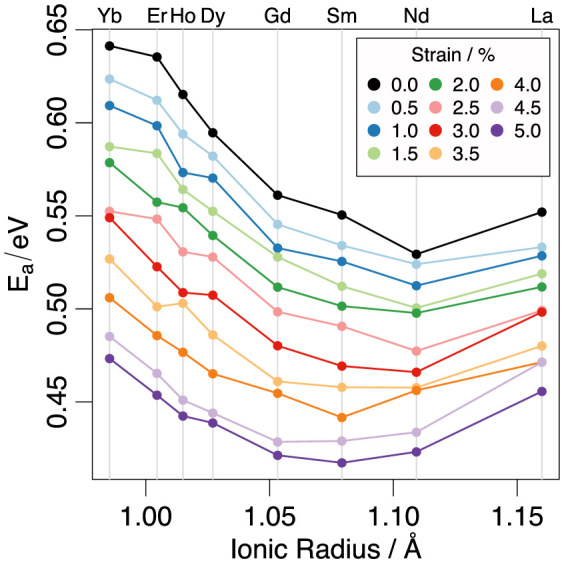
The dependence of the activation energies of oxygen migration on the dopant ionic radius and strain for Ce_0.9_R_0.1_O_1.95_.

**Figure 4:**
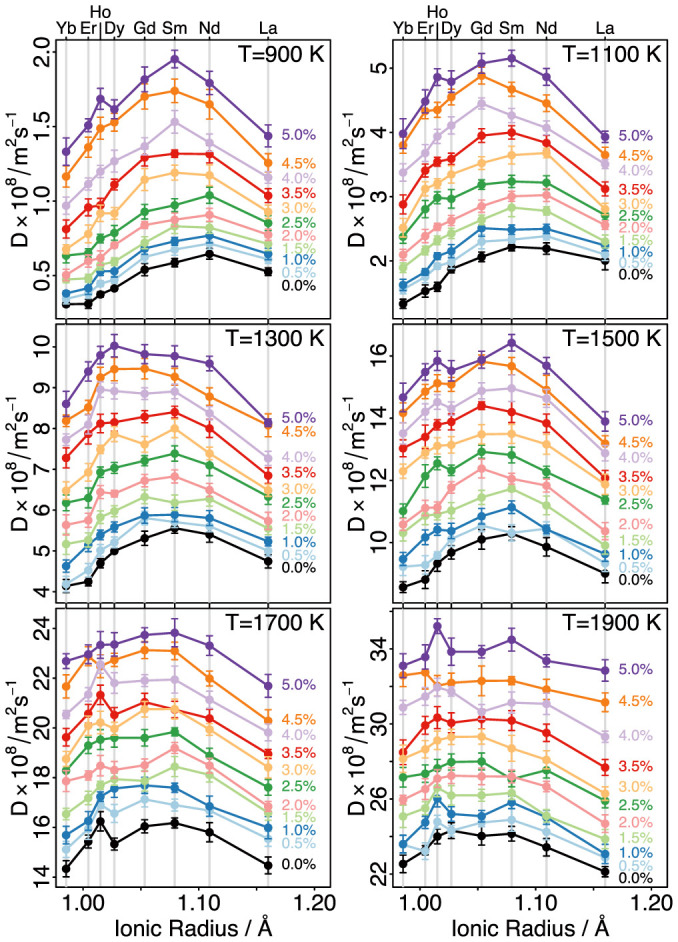
The dependence of the oxygen diffusivities on the dopant ionic radius and strain.

The black line in Figure 3 represents the activation energies of oxygen migration with respect to the dopant ionic radius in unstrained ceria. From the present results and the given dopant composition it is calculated that Nd-doped ceria has the lowest activation energy of oxygen migration. The low activation energy of migration for Nd-doped ceria is also reflected on the diffusivities for temperatures in the range 900–1500 K (refer to black line of Figure 4).

### Impact of strain

The dependence of the activation migration energies (Figure 3) and oxygen diffusivities (Figure 4) on strain was investigated in order to show any trend relating their magnitude to dopant size and uniaxial strain. Both Figures 3 and 4 show that for all dopants, a tensile strain brings about a decrease in the activation energy of migration of between 0.052 and 0.089 eV (for 2.5% strain) to 0.096–0.182 eV (for 5% strain). Systems containing smaller dopants (such as Yb) are more sensitive to the effects of strain as compared to the larger dopants (such as La). Figure 3 reveals that at uniaxial stains up to 2.5% Nd-doped ceria has the lowest activation energy of oxygen migration (0.48 eV), whilst above 3% the minimum in the E_a_ vs ionic radius curve shifts towards smaller ionic radii. Most notably there is a clear minimum for Sm-doped ceria for a strain of 4%. As strain increases beyond this level, however, this minimum becomes less clearly defined as the E_a_ for the Gd, Sm and Nd dopants becomes increasingly similar with values of 0.421 eV, 0.417 eV and 0.423 eV respectively, at a strain of 5%.

The importance of strain on rare-earth doped ceria is even more vividly expressed by the diffusivity values shown in Figure 4. These change significantly with respect to strain, which is consistent with the decrease in activation energy under tensile strain. Previous studies^19^ have suggested that conductivity may be increased by several orders of magnitude at strained interfaces between dissimilar oxides. Based on the results of the present study we calculate that, although considerable, the increases in oxygen diffusivity due to strain are not of the same order as these previous results may suggest (although it should be noted that biaxial conditions would be expected at such interfaces rather than the uniaxial conditions described here).

With the increase in temperature and concomitant increase in diffusivity, there is another trend that can be observed: the difference in diffusivities between dopants is decreased. This can be explained by the diminishing importance of the association between the dopants and the migrating oxygen vacancies as the T is increased. In essence, at high temperatures (above about 1000 K) the activation energy of migration of oxygen vacancies will be given by the energy barrier required for motion and will not be dependent upon the association energy of defect clusters formed between oxygen vacancies and the rare-earth dopants^42^,^43^. The association energy relates to the Coulomb interactions (favouring nearest neighbour cluster configurations) and the relaxation of the ceria lattice (favouring next nearest neighbour cluster configurations)^44^. Tensile strain will lead to the lowering of the association energies of clusters formed between the rare-earth dopant atoms and oxygen vacancies (whereas under compressive strain the association energies increase)^25^. The decrease of the association energy affects smaller dopants (Yb, Er, Ho, Dy) to a larger extent than the larger dopants (Nd, La), as they are more bound under unstrained conditions^44^. At increased temperatures there is a “flattening” of oxygen diffusivities in Ce_1-x_R_x_O_2-x/2_ (mainly due to the reduction of the association energies of the intermediate dopants: Gd, Sm) and this is nearly independent of the applied tensile strain.

**Figure 5:**
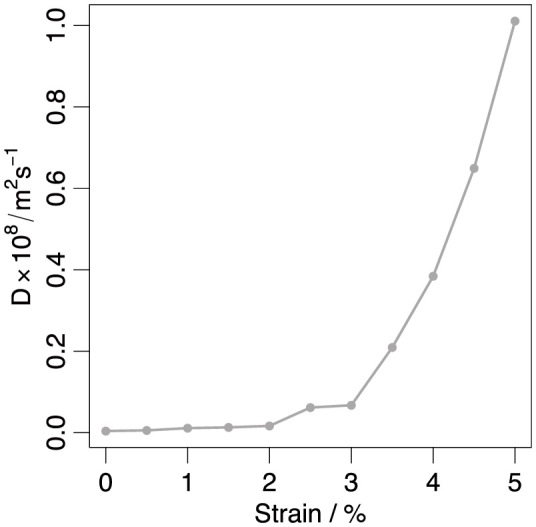
Diffusion coefficient oxygen self-diffusion in undoped ceria as a function of strain at T = 1900 K obtained from the average of 5 × 5 ns duration MD simulations.

Oxygen vacancy-dopant binding can only partly explain the increase in diffusivity under applied strain however, Figure 5 shows the trend in D for undoped ceria as a function of tensile strain. As for the doped cases, D values were obtained by performing a straight line fit to mean squared displacement versus time data. As the diffusion rate is much lower in the undoped case, the data in Figure 5 was collected over a longer production time of 5 ns, in order to enable statistically significant results to be obtained, even though a relatively high temperature (1900 K) was employed, furthermore the D values within Figure 5 were averaged over five such runs. For strains below 3% the D value obtained from the simulations was very small and showed no clear dependence on strain. Above this strain level, however, D increased from 6.7 × 10^−10^ m^2^s^−1^ to a maximum of 101.1 × 10^−10^ m^2^s^−1^ as the strain increased from 3.0% to 5.0%. This represents an increase of ~15 times.

As described above within doped ceria, increases in diffusivity due to strain can be attributed to a strain induced decrease in dopant-oxygen vacancy association. The results in Figure 5 are notable as they suggest that, even in the absence of such dopant-vacancy binding effects, an applied strain can lead to meaningful increases in diffusion rates. Assuming that similar mechanisms operate in both doped and undoped cases, a substantial proportion of the increase in doped ceria's diffusivity resulting from strain may be due to effects other than dopant-vacancy binding, especially at large strains.

**Figure 6:**
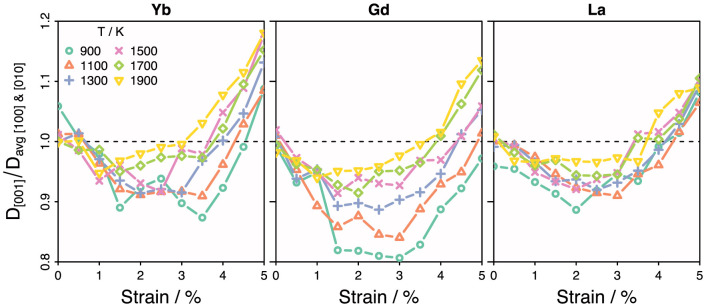
Oxygen diffusion coefficient along strain direction (D_[001]_) divided by that perpendicular to applied strain plotted as a function of strain. Isotropic diffusion occurs when this ratio is 1.0. When D_[001]_/D_[100]&[010]_ < 1.0 diffusion is higher in *ab* plane whilst values greater than unity indicate preferential diffusion along the direction of strain (*c* axis).

The uniaxial strain applied along [001] leads to anisotropic oxygen diffusion rates. Examination of the oxygen trajectories obtained during the data-collection stage of the MD simulations showed that diffusion occurred via a hopping mechanism. For both strained and unstrained cases, hops occurred between oxygen sites parallel to the [100], [010] and [001] directions. Whilst this behaviour persisted as the system was strained, the relative magnitude of the oxygen mean squared displacements along the [001] strain direction, changed in comparison to the [100] and [010] directions (which remain symmetrically equivalent even in the strained systems). This is demonstrated in Figure 6, in which the ratio of D_[001]_ (the oxygen diffusion coefficient along strained direction) to the average of the diffusion coefficients perpendicular to the applied strain (D_[100]_ & D_[010]_) are plotted as a function of strain for three dopants. The dopants Yb, Gd and La were chosen as these span the entire range of dopant radii examined; with Yb being the smallest, La the largest and Gd having a radius intermediate between the two.

Within the uncertainty of the simulation results, Figure 6 shows that when ε = 0 diffusion is isotropic as D_[001]_/D_avg [100] & [010]_ = 1.0. When strain increases, however, all three dopants initially show a decrease in this ratio, which then through a minimum before increasing again. For the Yb and La systems, D_[001]_/D_avg [100] & [010]_ < 1.0 for all temperatures considered in the strain range 0–4%. When the D ratio is below unity this indicates that diffusivity was higher in the plane perpendicular to the applied strain. Within the Yb and La doped systems, when strain was above 4%, the ratio switched to become larger than 1.0 at all simulation temperatures. This suggests a change in behaviour whereby diffusivity becomes higher parallel to the strained direction (along the simulation box *c* axis) rather than in the *ab* plane as seen for low and intermediate strains.

The anisotropy of oxygen diffusion is a little different in the Gd doped system: the lower temperature curves (900 and 1100 K) do not show the distinct transition from *ab* plane diffusion to *c* axis diffusion. In particular the 900 K D ratio remains below 1.0 even at 5.0% strain, whilst the 1100 K curve is only slightly positive at 5% strain meaning the diffusion is effectively isotropic at this strain and temperature. Above 1100 K, Gd shows similar anisotropy to the Yb and La doped systems.

In summary, the present study has considered the impact of uniaxial strain and doping on oxygen diffusion in rare-earth doped CeO_2_ using dynamic atomic scale computer simulation techniques. The potential model presented reproduces the thermal expansion coefficients and the variation of the lattice parameter with respect to the dopant concentration. As the temperature increases the difference in diffusivities between the rare-earth dopants is decreased and this can be traced back to the diminishing importance of the association between the dopants and the migrating oxygen vacancies. By examining undoped ceria we identify also a complementary enhancement of the diffusivity due to the impact of the imposed strain on the diffusion processes.

## Methods

### Model

Within this paper, the effect of uniaxial strain on doped ceria compositions was examined using MD simulations. The details of the simulation method are now given. MD allows the trajectories of an atomic configuration to be predicted with time by considering the forces between them^45^,^46^,^47^. As a result the description of the interatomic forces is key to the method's success.

Whilst the CRG model includes many-bodied, electrostatic and pairwise components within its description of the O-O and Ce-O interactions, the dopant-oxygen interactions presented here, are somewhat simpler, including only long-range electrostatic and short-range pair-potential contributions. This latter short-range pair contribution (V_short_) was described using the Born-Mayer potential form^48^, such that a pair of ions, i and j, separated by a distance of r_ij_ have an energy given by: 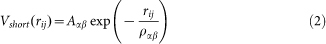
The parameters A_αβ_ and *ρ*_αβ_ are used to tailor the short-range potential to specific pairs of interacting species α and β.

The interactions between the trivalent dopants and oxygen ions have been derived specifically for this work using an empirical fitting procedure. The A_αβ_, ρ_αβ_ and C_αβ_ parameters for each R^3+^-O^2−^ interaction were obtained using an iterative procedure whereby the Nelder-Mead simplex minimisation algorithm^49^ was used to gradually adjust potential parameters in order to improve the match between the thermal expansion data of Pikalova *et al.*^36^ and the MD lattice parameters given by the potentials. At each iteration lattice parameters were obtained by equilibrating an 8 × 8 × 8 Ce_0.8_R_0.2_O_1.9_ super-cell for 10 ps in the NPT ensemble at temperatures of 300, 600 and 1200 K. By averaging cell size over the final 2.5 ps of these runs it was possible to obtain an objective measure of how well the MD predictions matched Pikalova *et al.*^36^ experimental data at each iteration of the simplex algorithm over the wide temperature range employed. Using this method a potential set was obtained that gives an excellent level of agreement with the experimental thermal expansion curves (a comparison between model predictions and experimental data is given in the results section), and potential parameters are given in Table 1.

Electrostatic interactions were calculated using the Particle-Particle Particle-Mesh (PPPM) solver based on the method of Hockney and Eastwood^50^ and implemented within the LAMMPS simulation code^51^,^52^. In line with the partial charges employed by the CRG model^29^, trivalent dopant ions were assigned charges of +1.6656e and Ce and O had charges of +2.2208e and −1.1104e respectively. A short-range potential cut-off of 11Å was used throughout. In addition the MD calculations employed periodic boundary conditions.

All simulation boxes were based on an 8 × 8 × 8 CeO_2_ super-cell. To obtain the desired dopant concentration trivalent dopant ions are substituted on to Ce sites at random with an oxygen vacancy introduced for every two dopant ions, by the random removal of an oxygen atom from the simulation cell (see equation 1). For the x = 0.1 cells this results in a cell containing the following atom totals: R^3+^ × 204, Ce × 1884 and O × 3994 (i.e. V_Ö_ × 102).

In order to examine its on oxygen migration it was necessary to apply tensile strains of between 0–0.05 to the CeO_2_. For each combination of dopant and temperature, strained systems were generated for each 0.005 strain increment within this range. Before applying these strains each system's unstrained equilibrium dimensions were determined.

Systems were initially equilibrated in the NVT ensemble for 2.5 ps (using a Berendsen thermostat^53^ to quickly establish the desired temperature) before Nosé-Hoover NPT dynamics were employed over 100 ps in order that the simulation box could relax to the size appropriate to the applied simulation temperature with no applied strain^54^,^55^,^56^. This relaxation procedure was performed with no applied pressure (i.e. the barostat pressure was 0GPa).

The zero strain condition obtained in this manner established the starting point for the strain ramping method, which is now described. Strain was imparted to the system by stretching the simulation box along the [001] direction such that after a 1 ns molecular dynamics simulation, a strain of 0.05 (5%) had been achieved. The length of the simulation box aligned along [001] was updated every 100 fs such that a true strain rate of 0.05 ns^−1^ was obtained.

Extending the system along the [001] direction results in a decrease in the system dimensions along the [100] and [010] directions (reflecting the system's Poisson's ratio). In order to accurately capture this effect, the simulation barostat was used to hold the [001] axis length at the value determined by the strain ramp whilst still allowing the other box dimensions and angles to vary in order to maintain a system pressure of 0 GPa. In this way the response of the system to the applied strain arising due to its Poisson's ratio was captured, as the x and y simulation box dimensions decreased as a result of the extension in z due to the tensile strain.

At each 0.005 strain increment encountered along the strain-ramp, the state of the system was stored, so that it could be used as the starting point for further simulations. On completion of the ramping method these snapshots were further equilibrated for 1 ns under NPT conditions, (with the [001] aligned cell length still being kept fixed). In this way the relaxed cell dimensions for each combination of dopant, temperature and strain increment were established.

Each data-point in the results given below has been averaged over seven distinct dopant configurations meaning that any bias introduced due to any individual dopant configuration is reduced. As a result, six simulation boxes, in addition to that obtained from the strain-ramp, were produced with different, random, dopant configurations. These were each scaled to give the box dimensions established during the strain-ramp but before performing the NPT equilibration described above. The seven simulation boxes then underwent a final equilibration and data-collection step, which is now described. The equilibration stage consisted of 20 ps of NVT dynamics, which was followed by 1 ns of NVE dynamics during which the system averages used in the results sections were obtained.

### Definitions

The activation energies of oxygen migration (or energy barriers) for the thermally activated oxygen diffusion is an important descriptor indicating how low the temperature the electrolyte can effectively function at. In the framework of transition state theory^57^, the oxygen ion exchange rate with vacancies, ν, along the migration paths can be described by the Boltzmann relationship as, 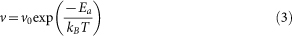
where *E*_α_ denotes the activation energy of migration and *v*_0_ is a constant. The oxygen vacancy diffusivity, *D*_v_, is connected to the mean square displacement <R^2^> of the oxygen vacancies via the Einstein relation, 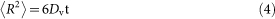
*D*_v_ exponentially depends on *E*_α_ similar to the formalism in eqn (3).

A way to quantify the interaction of point defects within the lattice is by the calculation of their association (or binding energies), *E_b_*, defined by 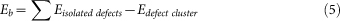
A positive binding energy implies that the defect cluster is more energetically favourable as compared to the constituent isolated defects.

## Figures and Tables

**Table 1:** Potential parameters for trivalent dopant ions

Interaction			*A*_αβ_/eV	*ρ*_αβ_/Å
Dy	-	O	36337.500	0.1901925
Er	-	O	35500.000	0.1873276
Gd	-	O	37562.031	0.1937708
Ho	-	O	36533.123	0.1886265
La	-	O	36197.135	0.2069536
Nd	-	O	37723.987	0.1994694
Sm	-	O	37224.275	0.1963756
Yb	-	O	33313.636	0.1854758
